# Thoracic Aorta Aneurysm Repair in a Patient with a Solitary Kidney: Hybrid Surgery as a Bailout Procedure

**DOI:** 10.3389/fsurg.2016.00062

**Published:** 2016-11-10

**Authors:** Chris Bakoyiannis, Demetrios Moris, George Karaolanis, Nikolaos Patelis, Dimitrios Schizas, Sotirios Georgopoulos, Theodoros Liakakos

**Affiliations:** ^1^Department of Surgery, Laikon General Hospital, Athens, Greece; ^2^Lerner Research Institute, Cleveland Clinic, Cleveland, OH, USA

**Keywords:** thoracic aorta, stent-graft, thoracic endovascular aneurysm repair, vascular access, hybrid surgery

## Abstract

Thoracic endovascular aortic repair (TEVAR) is an emerging treatment option for thoracic aorta aneurysms (TAA). Endovascular access is a challenge. We present a novel TEVAR technique in a patient with single kidney and a 6.4 cm TAA. Attempting to place a sheath through iliac arteries was unsuccessful. The decision to proceed to hybrid TEVAR was made. The protection of the solitary kidney was achieved through axillo-femoral bypass, followed by an end-to-side anastomosis between the aorta and a bifurcated graft. Through the graft, a stent was introduced in the thoracic aorta. With the use of contrast material, the right position of the graft was confirmed.

## Introduction

Thoracic endovascular aortic repair (TEVAR) is a relatively new and emerging treatment option for thoracic aorta aneurysms (TAA), and it would not be prefunctory to accept endovascular treatment as the approach of choice for many TAA patients in the years to come, even in an emergency setting (1).

However, several unresolved issues remain, in terms of anatomic restrictions and arterial access, and the need for further improvements and technological refinements will not cease any time soon (2). Achieving safe and successful endovascular access for introduction and deployment of the stent-graft device is a crucially important and often challenging step during TEVAR (2). The aim of this article is to present a novel TEVAR technique in a patient with single kidney.

## Case Presentation

A 70-year-old male was admitted at the emergency department with acute onset of severe back pain. The patient had a history of single functioning kidney, hypertension, diabetes mellitus, and grade 2 chronic obstructive pulmonary disease (COPD) due to heavy smoking. On physical examination during the episode, he was found to be hypertensive (BP = 169/99 mmHg) with a baseline creatinine value of 1.36 mg/dL. Of interest, the patient’s file included a radioisotope renography with Tc99m-diethylenetriaminepentacetate (DTPA), demonstrating 10% contribution of left kidney to total renal function.

**Figure 1:**
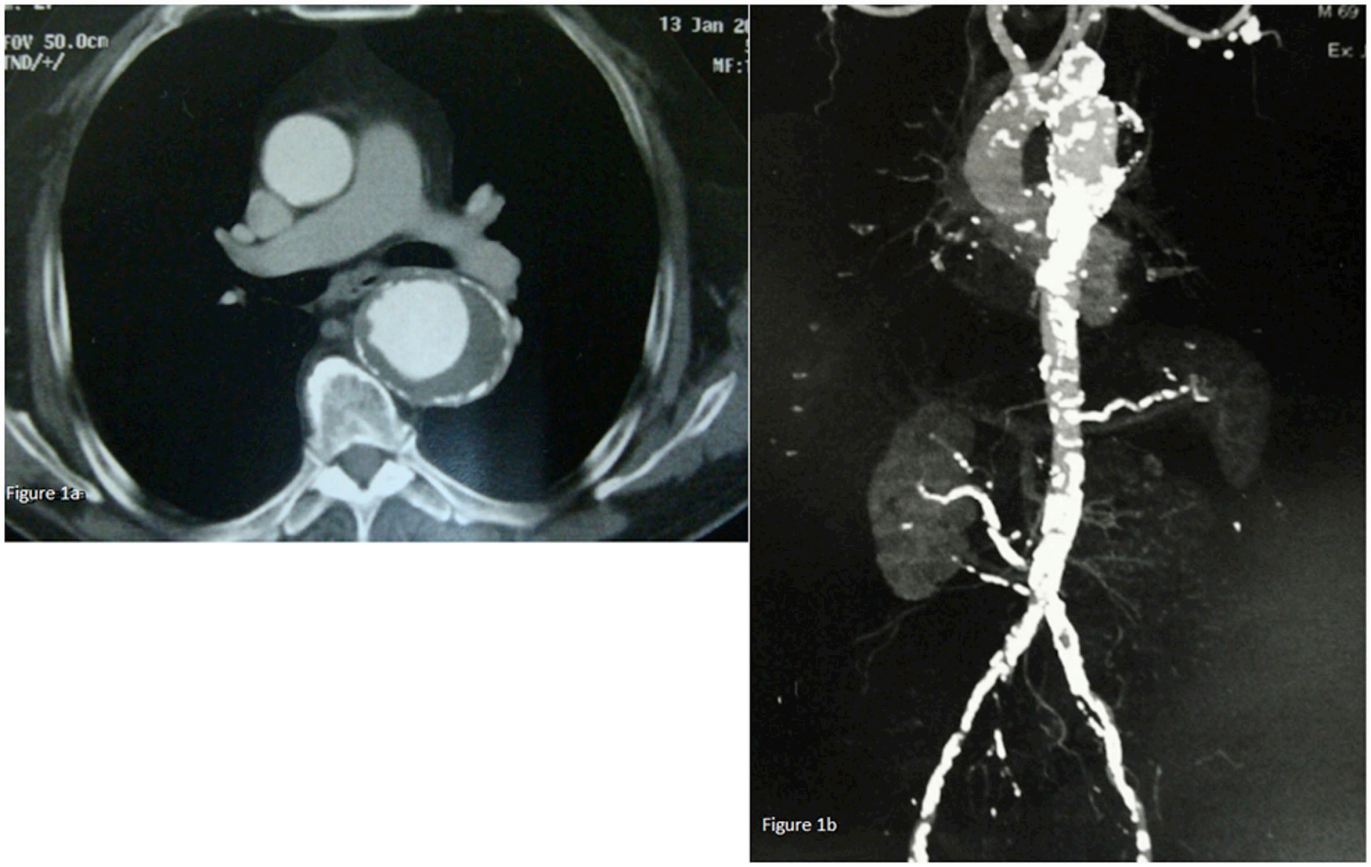
**(A)** Chest CT-scan revealing a thoracic aorta aneurysm with a diameter of 6.4 cm. **(B)** CT of the chest, abdomen, and pelvis revealing a thoracic aorta aneurysm extending 4.4 cm from the left subclavian artery and 10 cm from the abdominal aorta. Moreover, significant calcification of the abdominal aorta and the iliac arteries and stenosis of the right main renal artery whose origin was 2 cm above aortic bifurcation were also found.

A computed tomography (CT) of the chest, abdomen, and pelvis revealed a thoracic aortic aneurysm with a diameter of 6.4 cm, extending 4.4 cm from the left subclavian artery and 10 cm from the superior mesenteric artery (SMA) in the abdominal aorta. The length of the aneurysm was 8.1 cm, with proximal neck 3.3 cm and distal neck 2.8 cm (Figure 1A). Moreover, significant calcification of the abdominal aorta and the iliac arteries and stenosis of the right main renal artery whose origin was 2 cm above aortic bifurcation was also noted (Figure 1B). The patient was taken in the operative room for TAA repair.

## Procedure

Due to the stenosis of the iliac arteries, an attempt for iliac angioplasty was performed bilaterally. The attempts to place a sheath through the iliac arteries were unsuccessful. This was the main reason why the surgical team made the decision to proceed to the hybrid method of TEVAR.

The patient underwent a one-stage procedure under general anesthesia. A transperitoneal abdominal approach was used, allowing the exposure of the abdominal aorta, bilateral common iliac arteries (CIAs), and the origins of the renal arteries. The left renal vein was carefully mobilized, ligated, and divided by keeping adrenal, gonadal, and lumbar veins to improve access to the area and renal outflow intact. Once the exposure was completed, systemic heparin (7500 U) was administered.

**Figure 2:**
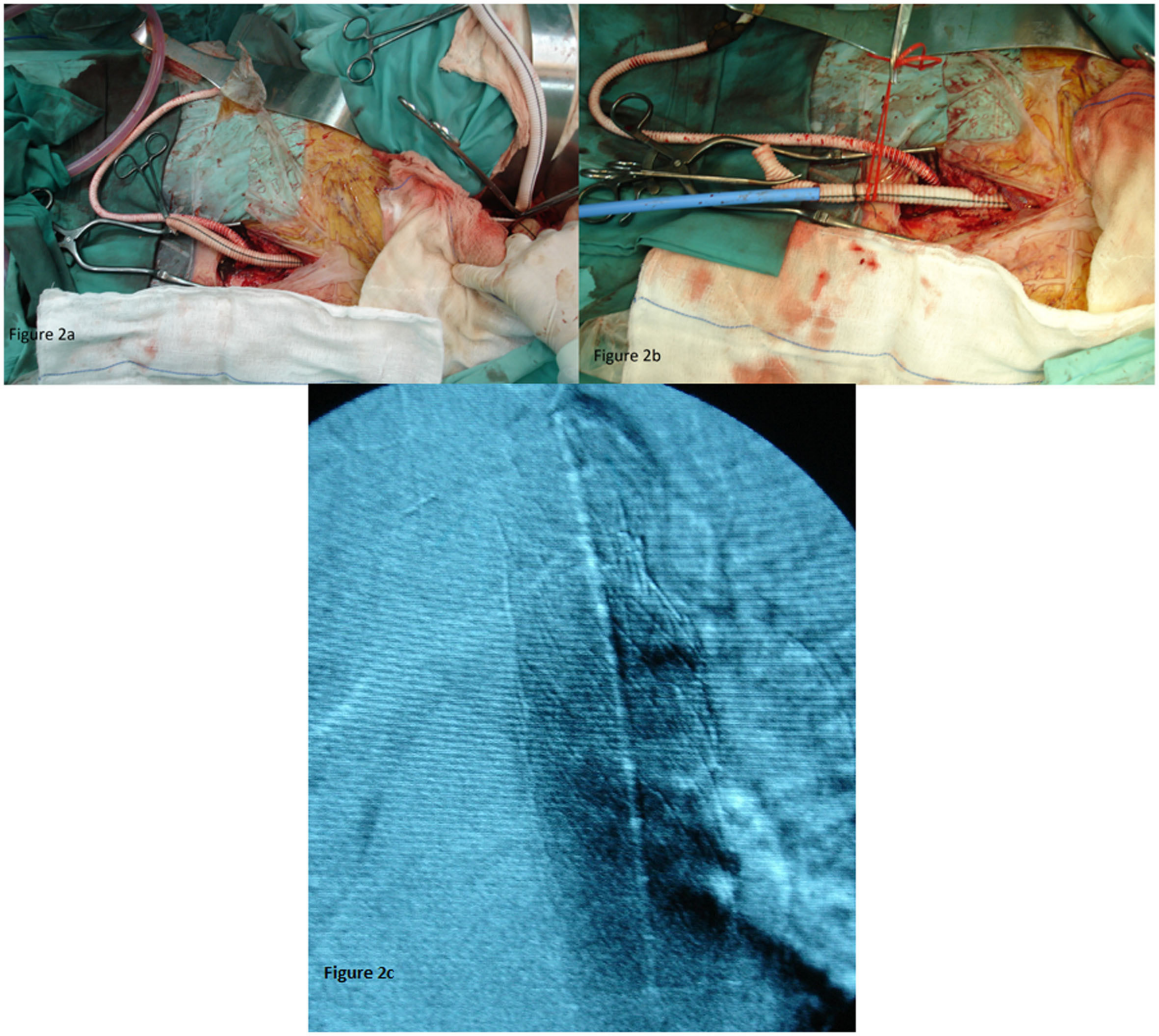
**(A)** The axillo-femoral bypass to ensure renal blood flow and the aortic bifurcated graft. **(B)** The aortic bifurcated graft as an access for TEVAR. **(C)** With the use of contrast material, the right position of the endograft was confirmed.

The protection of the functioning right kidney was achieved through an axillo-femoral bypass (Figure 2A). The retrograde flow from the axillary artery to the femoral and consequently in the renal artery after aortic clamping was important for the vitality of the kidney. An attempt to fix the stenosis of the right renal artery orifice was performed with endovascular means, but it was unsuccessful. With open approach, a reimplantation was thought as very risky since the vessel was extremely atherosclerotic. Afterward, an end-to-side anastomosis between the aorta and the bifurcated graft was performed (Figure 2A). The anastomosis was performed 5 cm below the SMA and roughly 2–3 cm below the imaginary orificies of the renal arteries, because lower aortic clamping was impossible due to extensive atherosclerosis (rocky tube). In addition, an angiographic catheter was placed through the left brachial artery in the thoracic aorta for better visualization of the stent placement.

Through the left branch of the graft, the sheath and the stent graft were introduced in the thoracic aorta (Figure 2B). We kept the amount of contrast used at as low as possible, the optimal position of the endografts (*n* = 2) with diameter 32 mm and length 10 cm (W. L. Gore & Associates) were confirmed (Figure 2C).

The last steps of the procedure included the removal of the axillo-femoral bypass and the performance of aorto-bifemoral bypass. The two limbs of the graft were anastomosed at the femoral arteries at the end of the procedure. The overall operative time was 4.5 h. The postoperative course of the patient was sound and the patient was discharged on 10th postoperative day with a creatinine value of 1.53 mg/dL.

## Discussion

This report adds to current knowledge and belief that combined surgical and endovascular repair of extensive aortic pathologies could be feasible and safe. The described procedure was designed for a patient with decreased pulmonary function and a single functioning kidney, a candidate to suffer from severe renal impairment during TEVAR. Additionally, due to severe atherosclerosis to common iliac and femoral arteries, the need for an alternative vascular access for endovascular repair was mandatory.

Without proper management, TAAs can worsen rapidly, with a rupture rate of approximately 50% (3–5). Although surgical repair modalities have been improved, they still carry high mortality rates (10–16%) and are associated with increased complication rates (3–5). Patients with TAA often carry concomitant medical problems such as hypertension, COPD, cardiac disease, and chronic renal insufficiency, which contribute to higher morbidity and significant perioperative mortality when offered open surgery and thus they could be ideal candidates for TEVAR (2).

Achieving safe and successful endovascular access for introduction and deployment of the stent-graft device is the cornerstone and often challenging step during TEVAR. Femoral artery is acknowledged as the standard vascular access for TEVAR, feasible in at least 70% of cases (2). Henretta et al. (6) showed that 30% of patients are found to have hostile iliac artery anatomy and/or atherosclerosis that may preclude transfemoral access. Our case is a typical example of impossible retrograde access for TEVAR and the main reason that led us to choose a hybrid technique as a bailout procedure.

Literature is still scarce on the feasibility, adequacy, and safety of endograft access alternatives, when femoral axis is hostile. CIA axis remains the first alternative for retrograde access by anastomosing a graft conduit to the CIA (2), offering technical simplicity and speed despite recent results showing higher rates of surgical complications and mortality (7). In our case, due to severe atherosclerosis, it would be difficult to perform an end-to-side anastomosis of the graft conduit to CIA, which would have provided us the necessary “smooth entry angle” for the large sheath.

Criado et al. (2) suggested that in cases, as ours, of unsuitable anatomy, significant calcified atherosclerotic disease, and previous endovascular and/or surgical procedures, where it may be truly impossible for retrograde endovascular access to the thoracic aorta, the use of an antegrade transcarotid access may constitute a viable alternative. The reason why this technique was not chosen in our case is that it still lacks validation in terms of safety and viability. In the same setting, Jim et al. (8) suggested that antegrade access through a 10-mm polyester graft anastomosed to the ascending aorta could be a viable and safe alternative in cases of impossible retrograde access for TEVAR. The disadvantage of this approach in our case would be the need of thoracotomy that would increase the severity of the operation in terms of surgical stress and need for intensive care unit as well as the possibility of major postoperative complications (9–11). The technique of “retrograde iliofemoral endarterectomy facilitated by balloon angioplasty” (12) or “internal endoconduit,” (13) which would have been another options in our case, could not take place since the attempts to place a sheath through the iliac arteries were unsuccessful.

So, taking all these limitations into consideration, the surgical team decided to perform an end-to-side anastomosis between the aorta and the bifurcated graft conduit. Consequently, through the left branch of the graft, first the sheath and later the stent graft was introduced in the thoracic aorta.

The novelty in our case was the presence of a single functioning kidney, with the orifice of the renal artery (right one) originating 2 cm above the aortic bifurcation. In order to ensure the appropriate blood flow to the kidney, an axillo-femoral bypass was performed with the retrograde flow from the axillary artery to the femoral and consequently in the renal artery after aortic clamping, being responsible for the vitality of the kidney.

Wang et al. (14) recently showed that patients with preoperative renal insufficiency maintain renal function after TEVAR. Despite the fact that 11% of them may require dialysis especially when risk factors such as emergency repair and baseline creatinine (>2.0 mg/dL) coexist.

## Conclusion

Achieving safe and successful endovascular access for introduction and deployment of the stent-graft device still remains as challenge during TEVAR. To the best of our knowledge, the current case is the first report of a TAA with single functioning kidney treated with TEVAR through an aortic graft conduit; technique that was shown to be safe and feasible.

## Ethics Approval and Consent to Participate

Written-informed consent was obtained from the patient for publication of this Case report and any accompanying images. A copy of the written consent is available for review by the Editor of this journal.

## Author Contributions

DM, GK, and NP designed the report; GK and DS collected the patient’s clinical data; CB, DM, and GK analyzed the data and wrote the paper, SG revised the draft, and TL supervised the manuscript.

## Conflict of Interest Statement

The authors declare that the research was conducted in the absence of any commercial or financial relationships that could be construed as a potential conflict of interest.
